# Scion on a Stock Producing siRNAs of Potato Spindle Tuber Viroid (PSTVd) Attenuates Accumulation of the Viroid

**DOI:** 10.1371/journal.pone.0057736

**Published:** 2013-02-28

**Authors:** Atsushi Kasai, Teruo Sano, Takeo Harada

**Affiliations:** Faculty of Agriculture and Life Science, Hirosaki University, Hirosaki, Japan; Friedrich-Alexander-University Erlangen-Nurenberg, Germany

## Abstract

Plants can attenuate the replication of plant viruses and viroids by RNA silencing induced by virus and viroid infection. In higher plants, silencing signals such as small interfering RNAs (siRNAs) produced by RNA silencing can be transported systemically through phloem, so it is anticipated that antiviral siRNA signals produced in a stock would have the potential to attenuate propagation of viruses or viroids in the scion. To test whether this is indeed the case, we prepared transgenic tobacco (*Nicotiana benthamiana*) expressing a hairpin RNA (hpRNA) of *Potato spindle tuber viroid* (PSTVd) in companion cells by using a strong companion cell-specific promoter. A grafting experiment of the wild type tobacco scion on the top of the transgenic tobacco stock revealed that accumulation of PSTVd challenge-inoculated into the scion was apparently attenuated compared to the control grafted plants. These results indicate that genetically modified rootstock expressing viroid-specific siRNAs can attenuate viroid accumulation in a non-genetically modified scion grafted on the stock.

## Introduction

Viroids are the smallest known infectious agents of plants, and induce disease in a wide range of hosts including many crop species. They have been identified as non-coding, circular, single stranded RNAs ranging in size from 246 to 401 nt [1]. Viroid replication is entirely dependent on transcriptional and processing machinery supplied by the host, and transport of the resulting progeny utilizes preexisting cellular pathways [2], [3]. On the other hand, exogenous RNA invader such as viroids induce RNA silencing system in the infected plants in which dicer-like (DCL) protein, argonaute (AGO) protein, and RNA-dependent RNA polymerases (RDR) function sequentially. As a result, high levels of viroid-specific small RNAs can accumulate in host plant cells [4]–[6]. However, viroids appear relatively resistant to RNA silencing, probably due to the highly conserved secondary structure of the genome RNA itself [7]–[9]. Meanwhile, transgenic tomato plants that accumulate high levels of viroid hpRNA-derived siRNAs (hp-siRNAs) exhibited effective resistance to PSTVd infection [10], [11]. Furthermore, Di Serio et al. [12] have reported that when the gene for RNA-dependent RNA polymerase 6 (RDR6), catalyzing an amplification circuit producing the double-strand precursors of secondary siRNAs, has been silenced, *Nicotiana benthamiana* plants accumulated increased amount of PSTVd genomic RNA comparing to the wild-type control, supporting the contention that viroid hairpin-derived siRNAs can reduce the accumulation of viroid by RNA silencing system.

RNA silencing by siRNAs in plants has the ability to spread systemically. This does not involve gradual cell-to-cell spread. The silencing signal travels along the vascular system and induces silencing in sink tissues [13], [14], was first demonstrated convincingly by a grafting experiment using *Nicotiana* plants. Furthermore, a specific siRNAs produced in squash stock has been detected in phloem sap from a grafted cucumber scion [15]. Additionally, much of the evidence that translocation of siRNAs induces systemic silencing has been based on grafting experiments [16]–[21]. These silencing signals travel to metabolic sink tissues in the direction of phloem flow [22]. It has also been found that the presence of a transgene is dispensable for the RNA degradation step in plants when the endogenous gene transcript over-accumulates above the level present in wild-type plants [23]. We have also shown that root stock in which siRNAs is produced exclusively in companion cells can induce silencing of an endogenous gene in the scion, even though the silenced area is limited to that along the phloem [24].

Therefore, virus and/or viroid-derived siRNAs transported from stock harboring the siRNAs producing system would be expected to counteract the accumulation of invader RNA in the scion. If this is indeed the case, resistance of non-transgenic scion to virus and/or viroid infection might be improved by grafting on genetically-modified rootstock. Here we present for the first time the evidence that viroid-specific small RNA signals supplied from genetically-modified rootstock expressing non-infectious form of hairpin viroid RNA can attenuate the accumulation of viroid RNA in the scion.

## Results and Discussion

### siRNAs in CoYMV:PSTVd *N. benthamiana*

**Figure 1:**
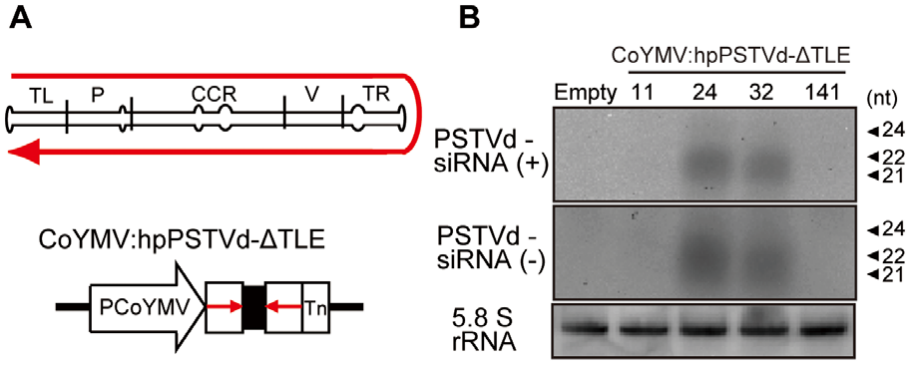
CoYMV:hpPSTVd-ΔTLE *N. benthamiana* plants. (A) Schematic diagrams of the CoYMV:hpPSTVd-ΔTLE construct. Arrows indicate PSTVd sequence used. pCoYMV; *commelina yellow mottle virus* promoter, Tn; nopaline synthase terminator, TL; terminal left, P; pathogenicity, CCR; conserved central region, V; variable region, TR; terminal right. (B) Northern blot analysis of PSTVd siRNAs in the transgenic plants. Small RNA enriched nucleic acid (10 µg) was analyzed in 15% polyacrylamide gel and probed with PSTVd negative (top) and positive (middle) strand RNA. 5.8S rRNA hybridization was used as a loading control.

The expression of a truncated, near full-length, hpPSTVd-ΔTLE was controlled by a companion cell-specific promoter, pCoYMV [25] (Figure 1A), to increase the potential siRNAs level in the phloem. The usefulness of pCoYMV for enhancing siRNAs signal in phloem transport had been confirmed in our previous studies [24], [26]. Since the promoter sequence was linked to near full-length PSTVd cDNA lacking 12 nucleotides at the left terminal end of the genome, the resulting construct (Figure 1A) facilitated the production of non-infectious form of hpPSTVd-ΔTLE sequence in the transgenic *N. benthamiana* companion cells.

Four CoYMV:hpPSTVd-ΔTLE integrated lines of *N. benthamiana* were obtained. Although a certain (ca. 5.3∼12.2%) proportion of the four T1 seedlings exhibited abnormal phenotypes such as dwarfism, delayed rooting, an aberrant leaf shape and so on, none of the T2 seedlings obtained exhibited any obvious phenotypical abnormalities. Lines 24 and 32 accumulated detectable levels of PSTVd-specific siRNAs by RNA-gel blot analysis, while the remaining two lines (11 and 141) did not show detectable levels of PSTVd-siRNAs (Figure 1B). As the integration of hpPSTVd-ΔTLE fragment into the *N. benthamiana* genome was confirmed by genomic PCR (Figure S1), the difference in the viroid siRNAs accumulation levels seemed to have been caused by position effect of the integration sites. As we have already reported that the production of siRNAs exclusively in companion cells ensured silencing of the target gene expression in the remote sink tissue [24], all the four transgenic lines including lines 11 and 141 with lower levels of PSTVd-specific siRNAs accumulation were also characterized in the following experiments.

### Effects on Expression of Specific Host Genes in CoYMV:hpPSTVd-ΔTLE *N. benthamiana*

To investigate whether the production of hpPSTVd-ΔTLE-derived siRNAs in companion cells affected the expression of endogenous genes, quantitative RT-PCR analyses of *NbBRP* (bromodomain-containing RNA-binding proteins 1 and 2, accession nos. AJ504729 and AJ504730), which are genes homologous to the tomato RNA-binding protein *SlVirp1* gene [27], were performed. Although it has been reported that *SlVirp1* mRNA is decreased in PSTVd-infected tomato leaves [28], the expression of both *NbBRP1* and *2* was inversely up-regulated in CoYMV:hpPSTVd-ΔTLE lines (Table 1). Furthermore, it was likely that a higher amount of PSTVd siRNAs led to higher *NbBRP* gene expression, notably in *NbBRP1*. Additionally, another gene, *NbAGO1* (*N. benthamiana ARGONAUTE1*), whose mRNA level was reported to have been increased by virus infection [29], [30], was analyzed. The results showed that the level of *NbAGO1* mRNA in hpPSTVd-ΔTLE lines was almost equivalent to that in control plants (Table 1).

**Table 1:** The expression levels of viroid responsive genes in CoYMV:hpPSTVd-ΔTLE plants.

Gene		CoYMV:hpPSTVd-ΔTLE			
	Empty	11	141	24	32
*NbBRP1*	92.90±15.12	98.12±7.21	121.76±33.35	172.89±20.52***	149.82±15.0***
*NbBRP2*	877.23±110.47	884.68±182.54	1029.34±212.35	1065.33±88.40	1161.01±214.91
*NbAGO1-1*	98.05±21.09	–	–	112.70±35.81	92.86±10.85
*NbAGO1-2*	105.89±14.43	–	–	114.07±30.68	86.55±7.11

The level of each gene expression was normalized based on *NbUbi*. One resulted value of *NbBRP1* and *NbAGO1-1* was based as a benchmark of 100, respectively.

– means no trial. Triple asterisk indicates significant difference (p<0.01) from the Empty.

Next, the transcription levels of possible target genes of small RNA of PSTVd, reported by Wang et al. [6], were investigated, since Wang et al. [6] and Navarro et al. [31] reported about the relationship between small RNAs of viroid and pathogenicity. We had already identified *N. benthamiana* genes that were homologous to those of possible target genes of small RNA of PSTVd in tomato. In fact, although the whole sequences of the tomato and tobacco genes showed high homology, they showed a few nucleotide differences within the possible target site (Figure S2A and B). Furthermore, in the case of SGN-U585132 (ARF GAP-like zinc finger-containing protein ZIGA3), the possible corresponding target sequence for tomato was not present in *N. benthamiana* (Figure S2B). The transcript of SGN-U580321 (NADPH:protochlorophyllide oxido-reductase POR) was slightly but significantly decreased in line 24, whereas the level of SGN-U567353 (phosphatase 2A) was unchanged.

Overall, it was suggested that PSTVd-specific siRNAs from the CoYMV:hpPSTVd-ΔTLE construct more or less affected the expression of the specific host genes, but the changing direction was not identical to that for natural PSTVd infection in tomato, or rather somewhat opposite in some cases [32]. The level of PSTVd-specific siRNAs accumulation in *N. benthamiana* infected with PSTVd is overwhelmingly higher than in plants transformed with CoYMV:hpPSTVd-ΔTLE. Furthermore, only phloem expression of siRNAs in CoYMV:hpPSTVd-ΔTLE plants showed relatively lower amount of siRNAs accumulation than in transgenic plants driven by 35S promoter. Therefore, the different responses observed in PSTVd-infected plants were not surprising. Also, to some extent, the different responses may have been attributable to the difference in target site sequences between tomato and tobacco and also the difference in the response to PSTVd infection; i.e, “Rutgers” tomato plant showed severe stunting and epinasty, however, *N. benthamiana* was totally a symptomless carrier for the PSTVd isolate. Furthermore, the difference of siRNA population between cases of viroid infection and CoYMV-hpPSTVd-ΔTLE expression might make the difference.

### Challenge Inoculation of Viroid into CoYMV:hpPSTVd-ΔTLE Lines

**Figure 2:**
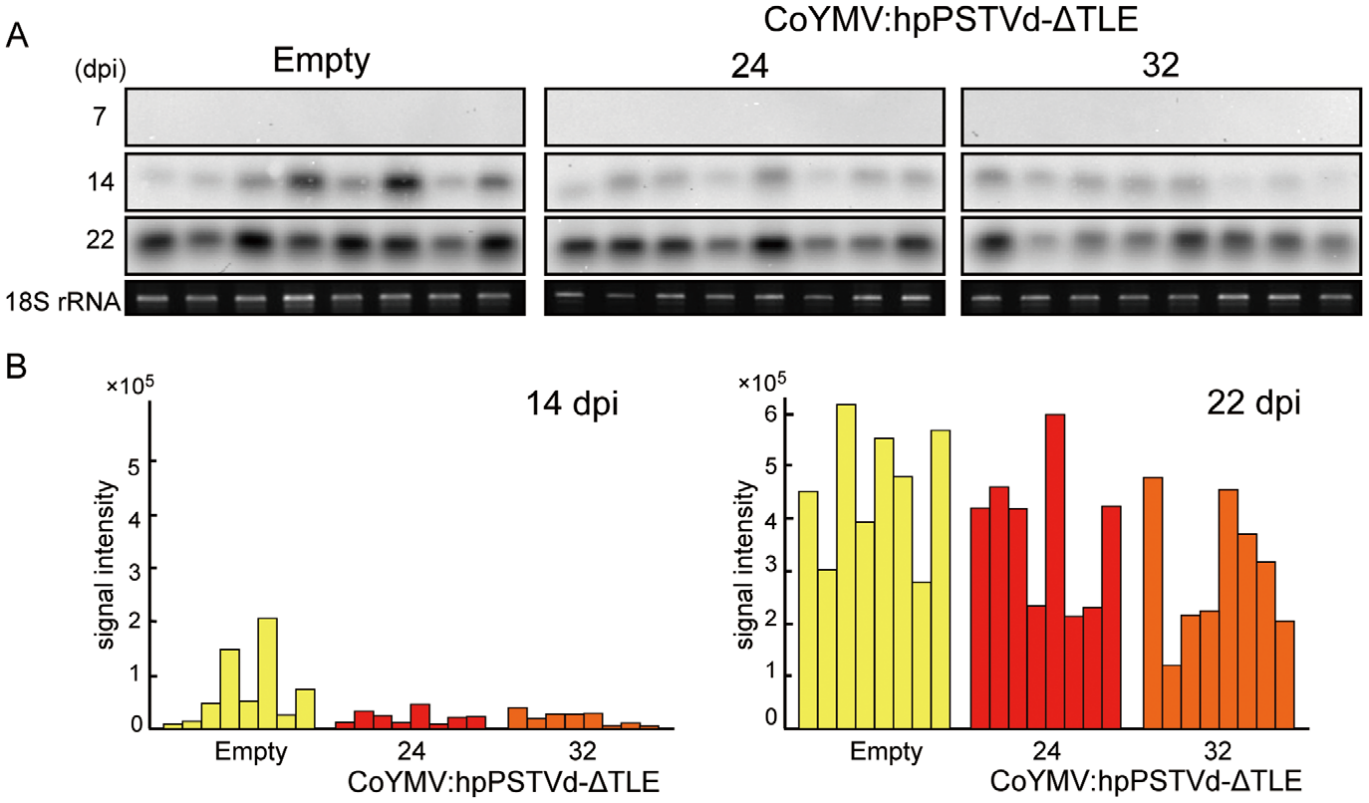
PSTVd accumulation in the CoYMV:hpPSTVd-ΔTLE lines. (A) Detection of viroid quantity by northern blot analysis of RNA extracted from the leaves of transgenic lines 24 and 32 at 7, 14 and 22 days post inoculation (dpi). Total RNA (100 ng) was hybridized with the PSTVd negative strand RNA probe. The results of 18S RNA qRT-PCR show equal loading of RNA. (B) Accumulated levels of the PSTVd RNA at 14 dpi and 22 dpi are shown diagrammatically. Quantity of total RNA was normalized against 18S rRNA. Note that hybridization of each dpi membrane was done for separate experiments.

**Figure 3:**
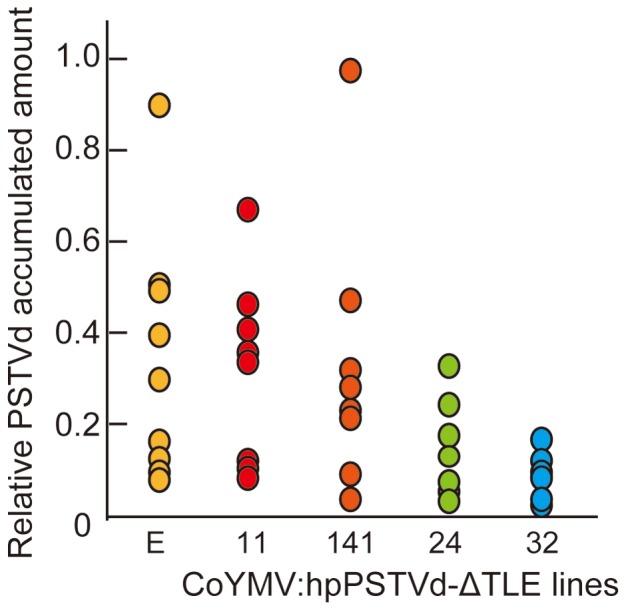
PSTVd accumulation in the CoYMV:hpPSTVd-ΔTLE lines at 14 dpi. The data were obtained by dot-blot hybridization (Figure S3) using 8 individual plants of each line.

To examine whether PSTVd accumulation can be attenuated in CoYMV:hpPSTVd-ΔTLE lines, we performed northern blot analyses to detect the mature PSTVd positive strand molecules in line 24 and 32 which accumulated detectable levels of the PSTVd siRNA by gel blot analysis (Figure 2). At 14 dpi, plants of all lines showed PSTVd accumulation. However, the signal intensities in lines 24 and 32 were weaker than those in the lines Empty control (Figure 2A and B). That is, PSTVd accumulation was suppressed in the two CoYMV:hpPSTVd-ΔTLE lines, in agreement with a previous report indicating that PSTVd resistance is correlated with the level of hpPSTVd siRNAs accumulation [11]. Although the period of attenuation was not prolonged and differences in the signal intensity between the lines were not observed at 22 dpi anymore, PSTVd accumulation was evidently restrained at 14 dpi. To confirm the attenuating effect of hpPSTVd-ΔTLE small RNAs on PSTVd accumulation, we performed another experiment using eight individual plants of CoYMV:hpPSTVd-ΔTLE lines including line 11 and 141 which did not show detectable levels of PSTVd-siRNAs (Figure S3). Dot-blot hybridization experiment was done as substitute for northern hybridization because of the concise analysis. As shown in Figure 3, although the levels of accumulation varied considerably within the same lines, all plants in line 24, and especially those in line 32, exhibited lower accumulation levels relative to the other three lines. Thus, it is appeared that PSTVd siRNAs production only in the companion cells was at least among the promising methods to attenuate PSTVd accumulation in the genetically modified plant.

### Attenuation of PSTVd Accumulation in the Scion Grafted on Genetically Modified Stock Containing CoYMV:hpPSTVd-ΔTLE Lines

**Figure 4:**
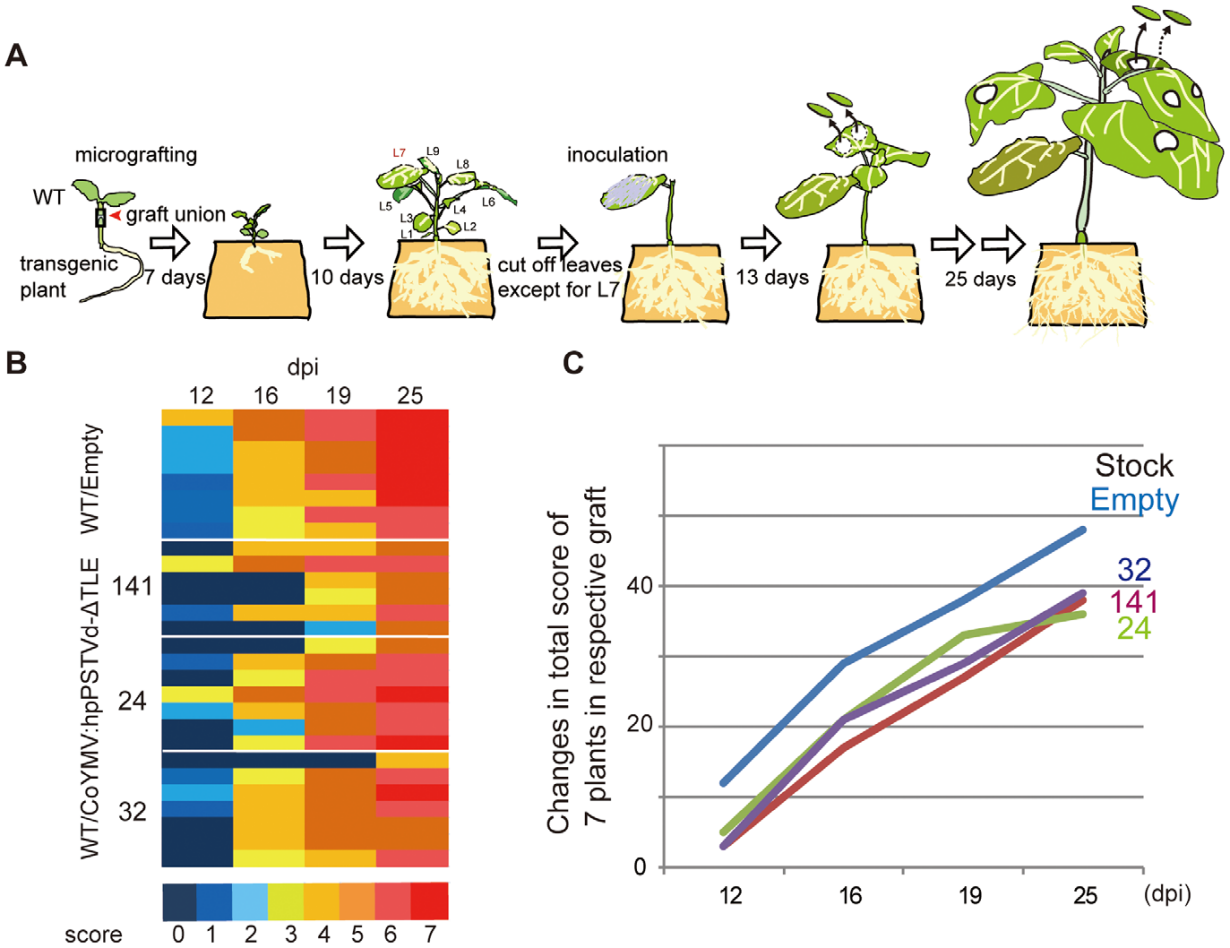
PSTVd accumulation in the WT scion on a CoYMV:hpPSTVd-ΔTLE stock. (A) Illustration of the experimental procedure. At 13 dpi, leaf disks were harvested from the most newly expanded leaf. Then, sampling was done every 3 days. Seven grafted plants per line were tested. Accumulation level of the PSTVd RNA was analyzed by dot-blot hybridization (Figure S4). (B) Levels of PSTVd accumulation in the WT scion presented in the form of a heat map. The intensity of the hybridization signals was normalized relative to the 5.8S RNA signal, and sorted out from 0 no signal to 7 maximal. (C) Changes in the accumulation levels of PSTVd. Total scores for 7 grafts in each line are represented.

Finally, to clarify whether PSTVd accumulation can be attenuated by a RNA silencing signals transferred from hpPSTVd-ΔTLE stock to the scion, we performed grafting experiments using wild-type *N. benthamiana* as the scion and CoYMV:hpPSTVd-ΔTLE lines as the stock. As shown in Figure 4A, to enhance the movement of the RNA silencing signals from the stock to the scion, the leaves of scion plants were removed except for one leaf, which was inoculated with PSTVd. At 12, 16, 19 and 25 dpi, the most newly expanded leaf at each time point was sampled for RNA extraction. All hybridization data were normalized by the 5.8S rRNA concentrations as the internal control (Figure S4), and then the levels of PSTVd accumulation in each plant were converted to a heat map (Figure 4B). At 12 dpi it was evident that 12 out of 21 wild type (WT) scions on the three lines of CoYMV:hpPSTVd-ΔTLE stocks gave no accumulation signs of PSTVd infection. Furthermore, even at 16 dpi, five out of a total 21 plants in the three lines of wild type WT/CoYMV:hpPSTVd-ΔTLE combinations continued to be negative for PSTVd, whereas all the seven plants in WT/Empty combination turned PSTVd-positive by 16 dpi. Furthermore, although all the plants showed PSTVd-positive at 25 dpi, the positive signals were somewhat dense in WT/Empty combination plants (Figure 4B). The changes in the levels of PSTVd accumulation from 12–25 dpi were quantified by using the sum of sores in the seven representative plants. As the result, it was apparent that all the three WT/CoYMV:hpPSTVd-ΔTLE combination plants attenuate PSTVd accumulation in the scions (Figure 4C). Interestingly, line 141, in which PSTVd-specific siRNAs accumulation was not evident (Figure 1), also attenuated PSTVd accumulation. As shown in Figure 3 and Figure S3, some of line 141 plants exhibited the attenuating efficacy of PSTVd accumulation. Therefore, line 141 is thought to have small amount level of the siRNA which was no detectable by northern blot analysis. Despite the lack of very clear efficacy of the siRNA transporting from stock to the scion, our observations have indicated that further research into the use of the siRNA producing stock as a mean of viroid disease resistant scion production is promising.

### Future Perspective

The results presented here provided a potentially useful technique for preventing plant from virus and/or viroid infections, in which we have constructed a hair-pin structure of near full-length (non-infectious form) of PSTVd and expressed under the control of companion cell–specific CoYMV promoter in *N. benthamiana* plants. Both of the genetically modified *N. benthamiana* plant itself and the wild type *N. benthamina* scion grafted onto the genetically modified *N. benthaniana* stock successfully suppressed PSTVd accumulation in the early stage of infection. The phloem transportation system to deliver RNA silencing signals from genetically modified rootstock to scion has already been succeed in an endogenous tobacco gene, glutamate-1-semialdehyde aminotransferase (GSA), in our previous study [24], and now the system is also promising for future improvement of plant resistance to virus and/or viroid infection. The improved scion variety resulting from transportion of RNA silencing signals such as siRNAs from the genetically modified rootstock does not contain the inserted transgene itself, meaning for examples that the fruits bearing on the scion are also free from transgene [33]. Thus there is an extensive merit in the system in associated with a possible risk of genetic contamination of ecosystem by pollen escaping to surrounding native vegetation either. It is anticipated that further application of stock delivering specific RNA silencing signals for scion improvement will lead to the development of rapid, easy, and environmentally systems for the improvement of crops, or particularly those such as fruit trees, in which grafting is commonly used for renewing the varieties [34].

## Materials and Methods

### Plasmid Construction

A truncated, near full-length, PSTVd-Intermediate strain sequence (PSTVd-ΔTLE; lacking the terminal left end nucleotides from No. 353 to No. 5 in the genome [35]), was amplified by PCR using primers tPSTVd sFw and tPSTVd sRv and subcloned into the *Eco*RV site of the pBluescript II SK+ plasmid (Stratagene, La Jolla, CA, USA). Then the CAT1 intron [36] amplified by PCR using primers intFw and intRv was integrated into the *Aat*II/*Hin*dIII sites of the plasmid. Finally, an additional PSTVd-ΔTLE sequence, amplified by PCR using primers tPSTVdaFw and tPSTVdaRv, was integrated into the *Hin*dIII/*Kpn*I sites, to make a hairpin (hp) structure of PSTVd-ΔTLE, in which a pair of PSTVd-ΔTLE is located in both boundaries of CAT1 intron by head to head orientation. The hpPSTVd-ΔTLE fragment from the plasmid was inserted into the *Bam*H/*Kpn*I sites of CoYMV-nos ter [24] based on pIG121 [37], and the resulting plasmid was termed CoYMV:hpPSTVd-ΔTLE. Construction of an empty vector as a control was done as described previously [24]. The DNA sequences of the plasmids were confirmed using an ABI PRISM 310 Genetic Analyzer (Applied Biosystems, Foster City, CA, USA).

### Plant Transformation and Growth Conditions

Transformation of tobacco (*Nicotiana benthamiana*) was performed as described previously [24]. The single functional T-DNA insert and the presence of the hpRNA structure were identified by 3∶1 segregation for kanamycin resistance in seeds obtained from the selfed primary transformants. Genomic DNA of each transgenic line was extracted using DNeasy (QIAGEN, Frankfurt, Germany) in accordance with manufacturer’s instructions. Genomic PCR analysis was performed using OneTaq DNA polymerase (New England BioLabs Inc., MA, USA). The primers used for genomic PCR are described in Table S1. Homozygotes for the integrated gene in 2-nd self progeny were confirmed by absence of antibiotic sensitivity. Two weeks after sowing, transgenic tobacco seedlings were transferred to rockwool (Nitto Boseki Co., Tokyo, Japan) in a standard nutrient solution (Ohtsuka House, Ohtsuka Chemical Co., Osaka, Japan) and grown in a plant growth room at 24°C under a 16-h light/8-h dark cycle with cool fluorescent light at about 100 µmol m^−2^s^−1^.

### Viroid Strains and Infections

*N. benthamiana* plants were mechanically inoculated with PSTVd. The inoculum contained 50 µg of PSTVd-enriched low molecular weight nucleic acid extract from tomato plants (*Solanum lycopersicum*, cultivar Rutgers) infected with PSTVd-Intermediate strain (accession no. AY937179) in 150 µl of 50 mM phosphate buffer (pH 7.0) containing 1 mg/ml bentonite. The 7-th true leaf of one-month-old plants were dusted with 600-mesh carborundum powder, and then gently rubbed with the inocula. Diethylpyrocarbonate-treated water was used for mock inoculation.

### Extraction of Small RNAs and Detection of siRNAs

Extraction of small RNAs and detection of siRNAs were performed essentially as described by Kasai et al. [24]. The digoxigenin-labeled truncated PSTVd sense and antisense riboprobes were synthesized using DIG RNA labeling Mix and T3, T7 RNA polymerase (Roche Diagnostics GmbH, Mannheim, Germany), respectively. The riboprobes were hybridized to small RNAs at 55°C in DIG Easy Hyb solution (Roche). The membrane was washed twice with 2×SSC/0.5% SDS at 55°C. The hybridized probe was visualized using CDP-star solution (Roche) and detected using a ChemiDoc XRS (Bio-Rad Laboratories, Inc., Hercules, CA, USA).

### Total RNA Extraction and qPCR Analysis

Total RNA was extracted using TRizol reagent (Invitrogen, USA), and treated with TURBO DNA-free (Applied Biosystems, Ambion, USA). In accordance with the manufacturer’s instructions, cDNA was synthesized with 1 µg RNA as a template using Superscript VILO (Invitrogen). The qPCR analysis was performed essentially as described in Kasai et al. [24] using SsoFast EvaGreen Supermix (Bio-Rad) with a Chrome4 realtime PCR detector (Bio-Rad). Information on the PCR primers for the *N. benthamiana BRP, AGO1, ZIGA3,* and *NAPDH* genes was obtained from the EMBL data base. The primers used for qPCR are described in Table S1.

### Dot and Northern Blot Hybridizations

The amount of PSTVd in *N. benthamiana* plants was assessed by blot analyses. Two discs (1.0 cm in diameter) were sampled from the upper most expanding leaves and subjected to extraction of the RNA fraction, as referenced [38]. One-microgram-RNA samples were diluted with denaturing buffer (0.5×SSC, 25% formamide, 8.75% formaldehyde) and denatured at 65°C. Then, the RNA solutions were spotted onto a nylon membrane (three spots per sample). For northern blot analysis, 1 µg of the RNA was separated in a 1.5% agarose gel containing 2.2 M formaldehyde and 20 mM 3-(N-morpholino) propanesulfonic acid (MOPS). The riboprobes were hybridized at 55°C in DIG Easy Hyb solution (Roche). The membranes were washed twice with 2×SSC at room temperature, with 2×SSC containing 1 µg/ml RNase A and then with 0.1×SSC containing 0.1% SDS at 65°C. Detection based on the hybridized signal was performed as described previously.

### Micrografting and Sampling from the Scion

Micrografting was performed essentially as described by Kasai et al. [24]. After 7 days grafted plants were transferred to rockwool (Nitto Boseki Co., Tokyo, Japan) in a standard nutrient solution. After 10 days of growth, the plants were stripped of all lateral leaves and buds except for the 7-th leaf and the shoot apex, and then these were inoculated with PSTVd inocula into the 7-th leaf. At 13 days post-inoculation (dpi), two leaf discs from the uppermost expanded leaf were sampled for RNA analysis.

## Supporting Information

Figure S1 **Genomic PCR of the CoYMV:hpPSTVd-ΔTLE lines.** (A) Schematic representation of each PCR fragment. (B) Genomic PCR analysis of four independent transgenic lines using three primer pairs. Quantity of genomic DNA was confirmed by *NbUbi*. Only IR3’ fragment was performed by 40 cycles and others were by 35 cycles.
(TIF)

Figure S2 **The expression of possible target genes of PSTVd-mediated RNA silencing in CoYMV:hpPSTVd-ΔTLE plants.** (A) Locations of potential mRNAs in tomato targeted by srPSTVd. (B) Alignment of possible srPSTVd target sites in tomato and *N. benthamiana* (left). Expression levels of three genes in transgenic tobacco whole plants harvested at one month after sowing. The levels of these mRNAs were analyzed by qRT-PCR. Error bars, s.d. (n = 3). **; significant difference from Empty (P<0.05). The amounts were normalized on the basis of *NbUbi*.
(TIF)

Figure S3 **Dot-blot analyses of PSTVd RNA in CoYMV:hpPSTVd-ΔTLE lines.** Transgenic lines were inoculated with PSTVd. At 7 and 14 dpi, leaf disks were sampled and the extracted RNA was dot-blotted. The membrane was hybridized with the PSTVd negative strand RNA probe. 5.8S rRNA was used as an internal control for the amounts of total RNA.
(TIF)

Figure S4 **Dot-blot analyses of PSTVd RNA in scions grafter onto respective stocks.** (A) Scion WT plants were inoculated with PSTVd (ca. 6.7 µg/20 µl) and the dot-blotted membrane was hybridized with the PSTVd negative strand RNA probe. (B) 5.8S rRNA was used as an internal control for the amounts of total RNA.
(TIF)

Table S1 Sequences of primers used in gPCR and qRT-PCR.
(TIF)
