# Supplementary figures and images for: Scion on a Stock Producing siRNAs of Potato Spindle Tuber Viroid (PSTVd) Attenuates Accumulation of the Viroid
Source: PLoS One. 2013 Feb 28;8(2):e57736. doi: 10.1371/journal.pone.0057736 (PMC3585205; doi:10.1371/journal.pone.0057736)

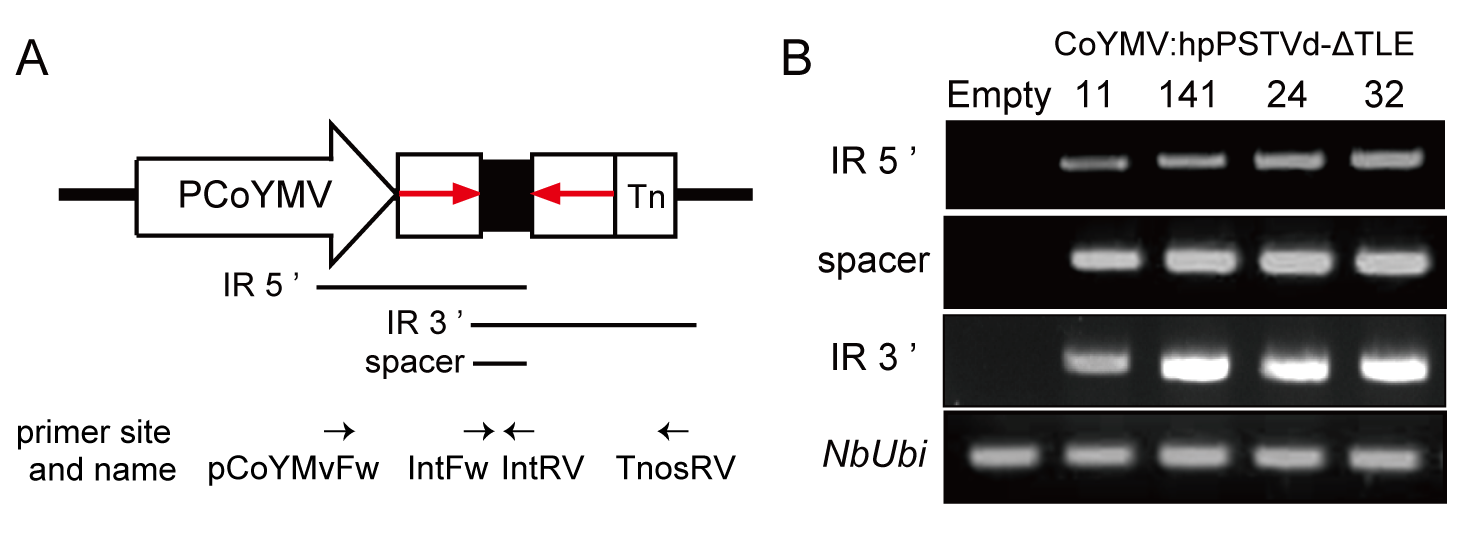

Supplement: Figure S1 — Genomic PCR of the CoYMV:hpPSTVd-ΔTLE lines. (A) Schematic representation of each PCR fragment. (B) Genomic PCR analysis of four independent transgenic lines using three primer pairs. Quantity of genomic DNA was confirmed by NbUbi. Only IR3’ fragment was performed by 40 cycles and others were by 35 cycles. (TIF) [file pone.0057736.s001.tif]

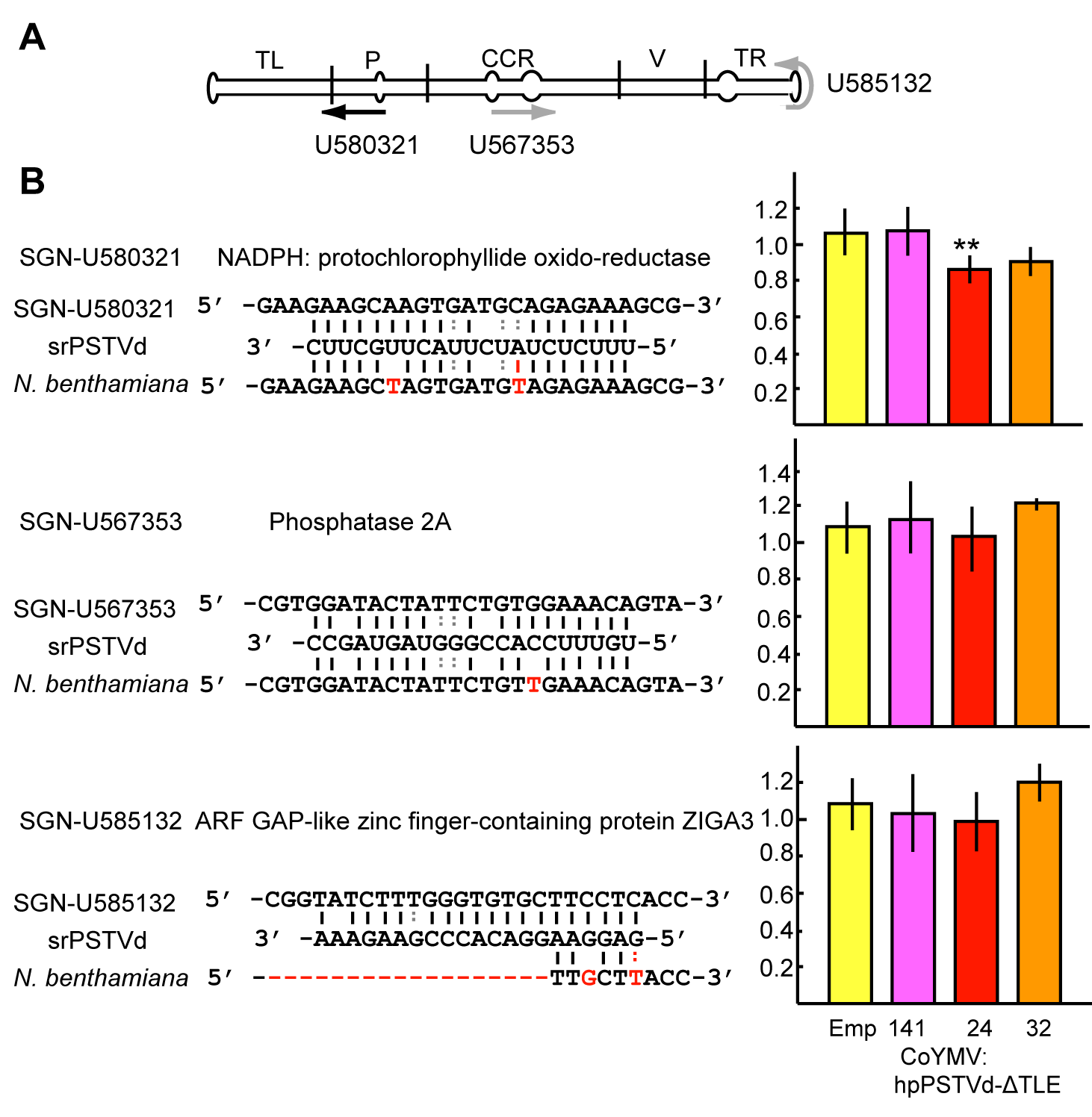

Supplement: Figure S2 — The expression of possible target genes of PSTVd-mediated RNA silencing in CoYMV:hpPSTVd-ΔTLE plants. (A) Locations of potential mRNAs in tomato targeted by srPSTVd. (B) Alignment of possible srPSTVd target sites in tomato and N. benthamiana (left). Expression levels of three genes in transgenic tobacco whole plants harvested at one month after sowing. The levels of these mRNAs were analyzed by qRT-PCR. Error bars, s.d. (n = 3). **; significant difference from Empty (P<0.05). The amounts were normalized on the basis of NbUbi. (TIF) [file pone.0057736.s002.tif]

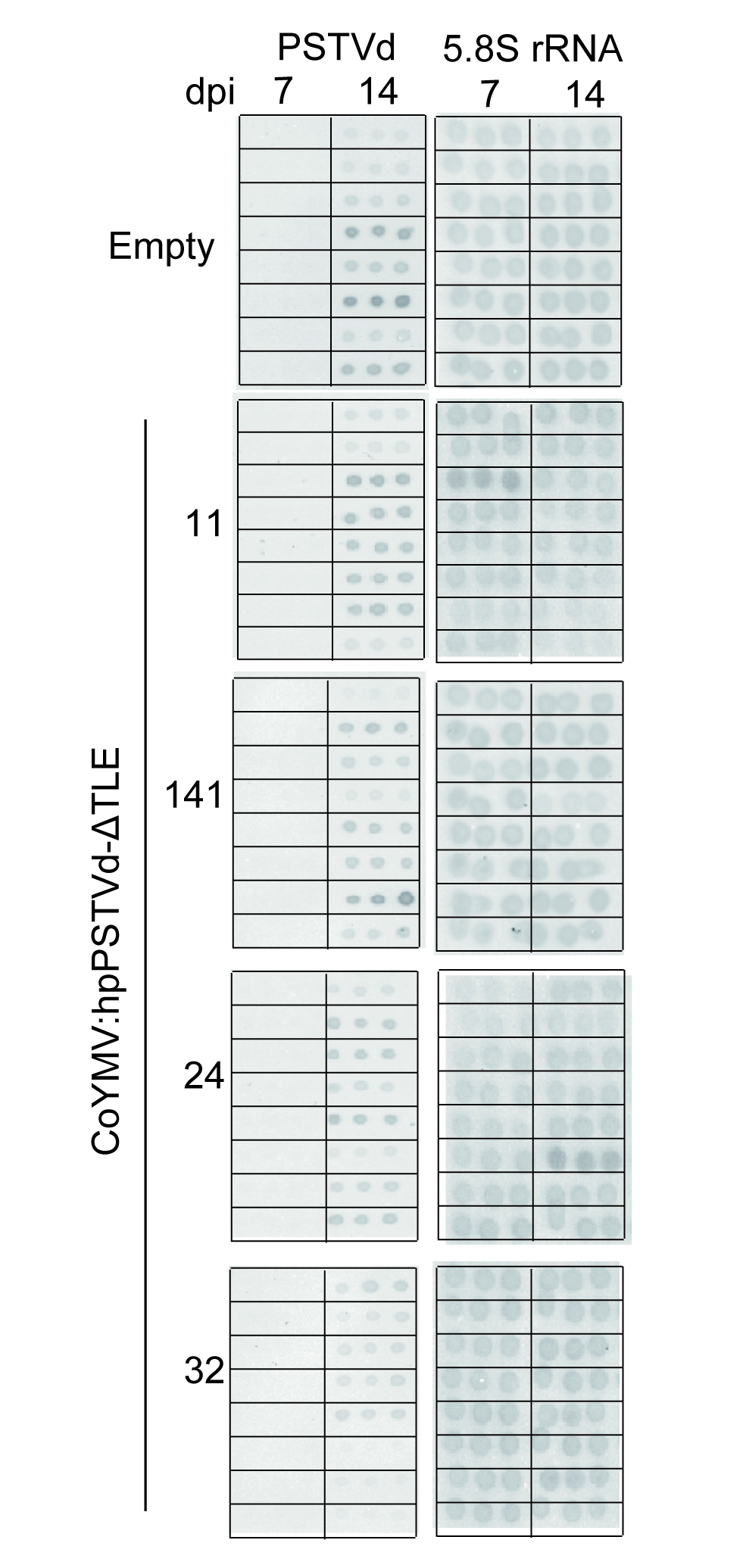

Supplement: Figure S3 — Dot-blot analyses of PSTVd RNA in CoYMV:hpPSTVd-ΔTLE lines. Transgenic lines were inoculated with PSTVd. At 7 and 14 dpi, leaf disks were sampled and the extracted RNA was dot-blotted. The membrane was hybridized with the PSTVd negative strand RNA probe. 5.8S rRNA was used as an internal control for the amounts of total RNA. (TIF) [file pone.0057736.s003.tif]

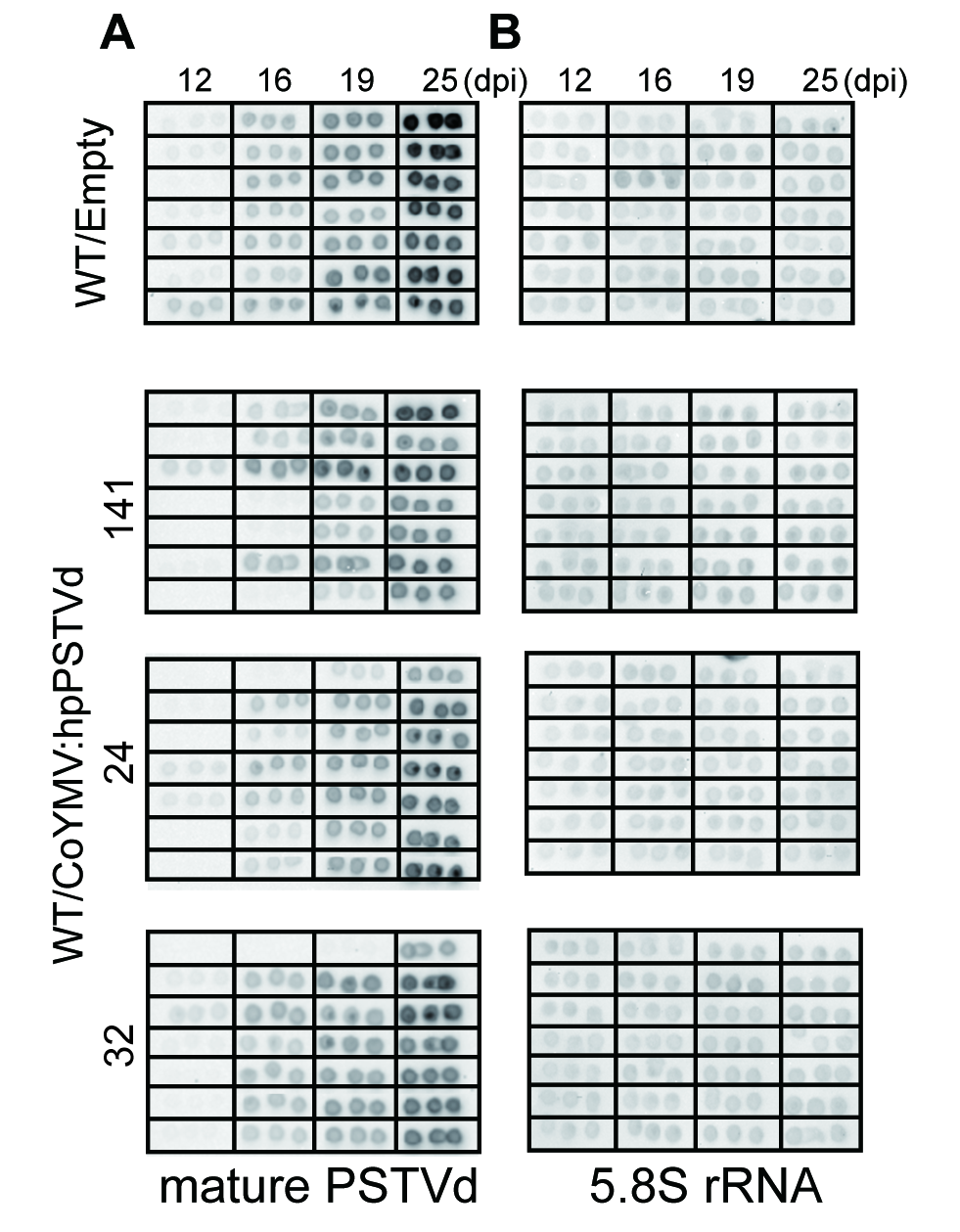

Supplement: Figure S4 — Dot-blot analyses of PSTVd RNA in scions grafter onto respective stocks. (A) Scion WT plants were inoculated with PSTVd (ca. 6.7 µg/20 µl) and the dot-blotted membrane was hybridized with the PSTVd negative strand RNA probe. (B) 5.8S rRNA was used as an internal control for the amounts of total RNA. (TIF) [file pone.0057736.s004.tif]

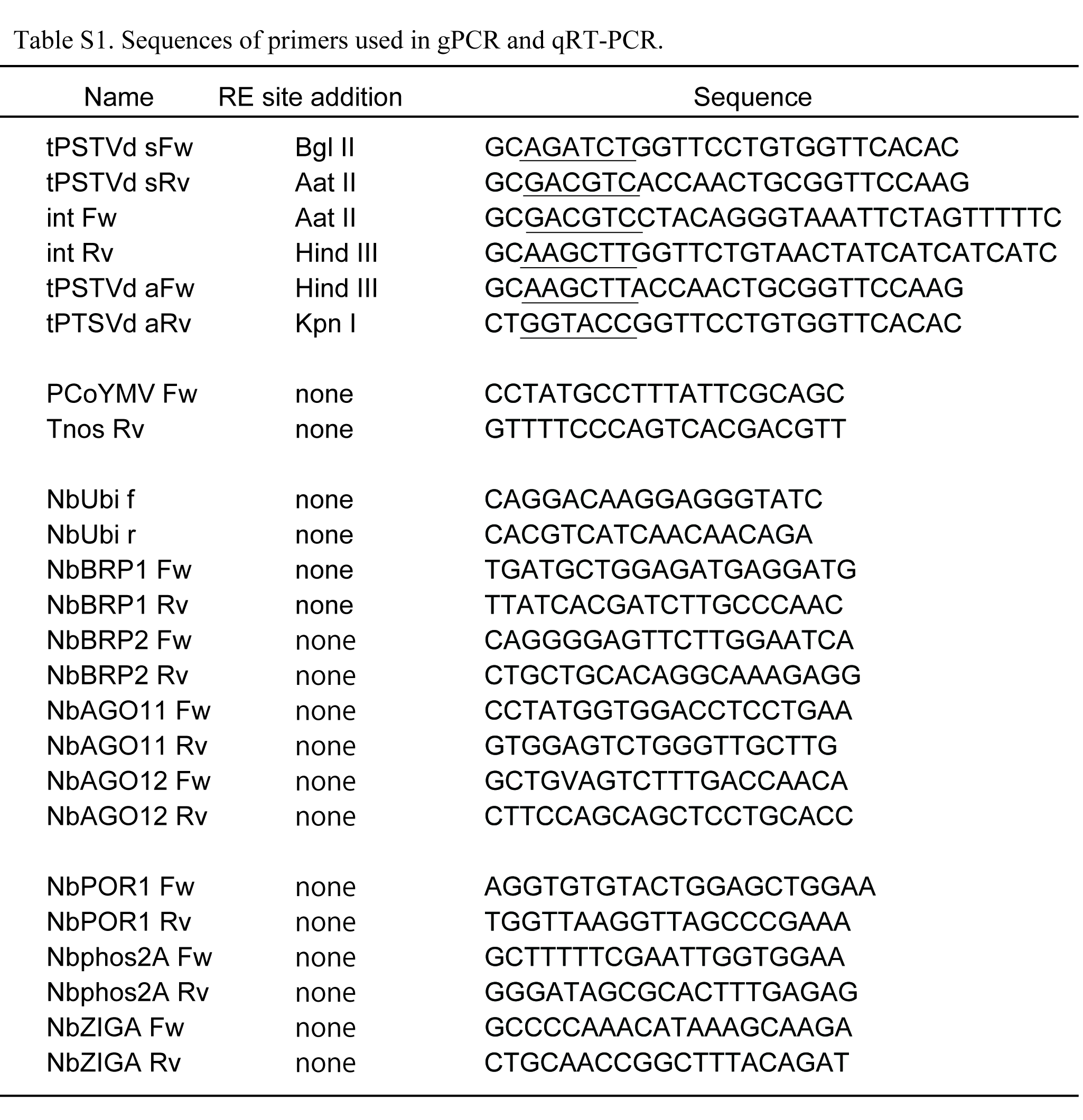

Supplement: Table S1 — Sequences of primers used in gPCR and qRT-PCR. (TIF) [file pone.0057736.s005.tif]
